# Foreign Summary

**Published:** 1840-05-11

**Authors:** 


### FOREIGN SUMMARY.

### PHILLIPS' LECTURE ON THE PRINCIPLES AND PRACTICE OF SURGERY.—NO. IV.

CANCER—(Continued.)

*Treatment continued—Cauterization—Excision.* MELANOSIS. — *Nature — Varieties — Fluid— Structure — Formation — Symptoms— Diagnosis—Prognosis—Causes—Treatment.* WOUNDS.— *General Principles — Varieties —* PUNCTURED: *Puculiarities — Prognosis — Treatment*—INCISED: *Peculiarities.*

*Cauterization.*—Many different modes have been employed in the use of cauterization; we may use the actual cautery or different forms of caustic—such as chloride of antimony, nitrate of silver, arsenic, caustic potash, nitrate of mercury, chloride of zinc, creosote. I think they are only applicable to cases where the disease affects the skin or mucous tissues alone; where the morbid structure, at its base, is not thick, and where two or three applications of the caustic are sufficient to destroy it entirely: they may sometimes repress vegetations, and determine upon the scirrhous base the formation of cicatrices. But these "cures" are only temporary; the ulcer will commonly reappear at the point where it was before seated. When more than one application of caustic is necessary, there is always danger that the irritation will give the tumour unwonted activity; therefore is it that it has been found most successful in those of the face; in the breast, or other gland, it should rarely be used, except in those cases where some portions remain after excision. Where caustics are admissible, many persons believe them preferable to the knife; it is said that relapses are less frequent after them. I object to the red-hot iron, because, if the surface be an irregular one, it cannot be applied to all parts of it, and because it too soon forms an eschar, by which its action is limited.

*Excision.*—The removal of cancer by a cutting instrument may be done by extirpating the cancerous mass alone, or by removing a limb upon which it is seated; and it is, no doubt, of all means the most efficacious. If it be judged necessary, it may be well that it should be preceded by leechings: the surrounding tumefaction is thus reduced, and the extent of the tumour more exactly defined; although the operation affords more chance of cure than any other, it is not easy to think that it justifies excision of the uterus, the rectum, &c. All operations are principally recommended from a belief of the occasional curability of the disease. A large number of persons regard it as incurable; and, to be consistent, the patient should be generally abandoned to the suffering of a horrible disease, and to inevitable death. It is, however, important, neither to deceive ourselves nor others with the results of operations. A case apparently the most simple, small, and circumscribed, completely removed locally, may rapidly reappear; whilst a very unpromising case sometimes does well. It is our duty to bring to bear all the resources of art, not to shrink from any difficulty, in attempting to rescue the patient from impending death; but we should hold out few hopes, nor encourage many fears, as to the results of such operations which we feel bound to undertake. In what cases, it may be asked, are we justified in operating—what in refusing? We shall be justified in hesitating, from the small prospect of success, when the tumour is large—when the neighbouring glands are tumefied, the surface ulcerated, and the constitution bears evident marks of suffering: but any of these circumstances taken singly may not be enough. The fundamental principle by which the surgeon should be guided, is the possibility of removing all the morbid structure. We may not succeed even when this is accomplished; we cannot, if it be not.

When no operation can with propriety be performed, we must seek to palliate suffering. The first element is diet; which should be rather vegetable than animal, and as little stimulating as possible. The bowels should be attended to, the patient should be warmly clad, and kept as free as possible from mental or bodily excitement. Pain may be lessened by opium and cicuta internally, by opiates and belladonna externally. The surface of an ulcer may be cleansed and purified by the chlorides, by decoction of bark, tincture of myrrh, alum, acetic or hydrochloric acid, charcoal, or carot, or yeast cataplasm. Hæmorrhages, which sometimes quiet pain, should be restrained if abundant. Pressure is sometimes enough for the purpose, but the red hot iron is sometimes necessary.

### MELANOSIS.

After Laennec, we understand, by melanosis, a pathological production deposited upon the surface or in the substance of organs, of a darkish or blackish colour, having no analogy with the healthy tissues of the body. How long this affection has been known, it is not very important for us to inquire: whether those passages in the works of Hippocrates, referred to by Bartholin and Lorry, can be admitted as indications that he knew the disease, is immaterial. I take it there can be no doubt that the case published by Highmore, in 1651, was melanosis. The disease which was described by Blugnoni, in 1781, which was hereditarily transmitted among the white horses of Chevasso, and which he termed hemorrhoids, was evidently melanosis; it was usually developed around the root of the tail and the anus. Some years later (in 1784,) the same disease was observed at Bresse. Gollety-Latournelle transmitted an account of it, in 1809: he says, "there supervened in a young stallion, on the second year of his covering, black "boutons," or buds, around the anus; they soon extended to the scrotum and sheath; they were placed between the skin and muscles, at first as large as a small nut; they increased until they attained the size of a pullet's egg; they did not supperate, and were insensible to the touch. In a short time, all the cellular tissue was similarly affected, and the animal died. When cut into, a matter like the grease of a cart-wheel flowed out. All the progeny of this stallion which had the same colour were similarly affected; those which were black, bay, roan, or iron-gray, escaped. In 1806, Laennec communicated to the faculty the result of his observations on the same subject. The subject was further elucidated by Breschet, in 1821; by Noack, in 1827; by Frousseau and Leblanc, in 1828; and by Carswell, in 1834; but much still remains to be done for it.

*Varieties.*—Laennec admitted two successive states in this disease; that of "crudity," and that of softening. In the first, it is a black, opaque, homogeneous mass, of the consistency of lymphatic glands; in the second, pressure causes the exudation of a darkish-red fluid, having very small blackish particles mixed with it; and when this softening is complete, it is converted into a black semi-fluid mass not unlike China ink, or the fluid of the cuttle-fish. It may occur in masses, surrounded or not by a cyst; may infiltrate an organ—may be deposited on surfaces—may be fluid, effused into cavities. When in masses, the tumour is variable in size; from that of a grape-seed to that of a hen's egg. Gohier saw one on a horse weighing thirty-six pounds: this might have been formed by the aggregation of many smaller ones. It is said that some of these tumours enlarge greatly, if the patient be placed in a bath. The colour of these masses appears, in many cases, to vary with the period of its development: at an early period they are of a reddish brown; they become darker, violet, very dark indigo, and sooty. The skin covering these tumours at first retains its natural thickness, but is gradually thinned, until only the epidermis remains, which, after a time, becomes dry, rugous, horny. With respect to the existence of a cyst at all, many doubts are entertained; some persons arguing that what is described a cyst is merely a condensation of the surrounding cellular tissue. This membrane, whether it be called a cyst or not, is, according to Breschet, together with the processes it sends into different parts of the tumour, the only organized part which he has observed; he could discover no vessel—no nerve; his injections were also arrested in this membrane, and did not penetrate into the black matter; but he has sometimes seen the injection extravasated with the black matter in the cells. The matter which constitutes these tumours when they are not infiltrations, varies much in consistency, from that of pitch to that of tar. If we rub it on linen or paper, in some cases, the tint is so like bistre that it may be used instead of it. Breschet says this matter is inodorous and almost tasteless. Noack, Gohier, Gasparin, and Flandrin, state that the odour is very sickly and disagreeable: this is my own observation; but I have only examined it on the dead body, and decomposition may have had something to do with it. Breschet says it is soluble in water and alcohol; exposed to the air, it putrefies very slowly. This is owing, Noack thinks, to the quantity of carbon it contains. The whole or part of an organ may be infiltrated with melanotic matter, which then fills up the cells or interstices of the tissue, but it is pretty certaln that many cases termed melanomia are chronic phlegmasia. Indeed infiltrated melanosis is very rare even in those cases where the disposition to produce the disease is very decided. It is occasionally found deposited upon certain surfaces, the peritoneum for instance, but oftener it will be found in the subserous tissue. When the black matter is not too concrete, the surrounding blood-vessels seemed filled with it. Breschet thought it was situated in the arteries; Noack thought it was especially found in the veins.

With respect to this melanotic fluid, some persons seem inclined to believe, that the black matter thrown up from the stomach in certain cases of acute or chronic inflammation of that organ was sometimes melanotic matter; it seems to me more probable that it was effused blood acted upon by the gastric fluid. Prout attributes to melanic acid the colour of urine in some cases where he has seen it of a deep black. The fluid is composed, after Jacquet, of water, carbon, iron, and phosphate of lime. Thenard believed it to be essentially formed of carbon; Laissaigné coloured fibrine, black colouring matter soluble in dilute sulphuric acid, and in a solution of subcarbonate of soda, (which reddens it) a little albumen, chloride of sodium, subcarbonate of soda, phosphate of lime, and oxide of iron; in fact, with the exception of the black colouring matter, not unlike the composition of the blood clot. Foy's analysis is as follows:

| | |
|---|---|
| Albumen, - - - - | 15.00 |
| Sub-phosphate of lime, - - | 8.75 |
| Water, - - - - | 18.75 |
| Fibrine, - - - - - | 6.25 |
| Hydro-chlorate of potash, - | 5.00 |
| ——— of soda, - - | 3.75 |
| Carbonate of soda, - - | 2.50 |
| ——— of lime, - - | 3.75 |
| ——— of magnesia, - | 1.75 |
| Oxide of iron, - - - | 1.75 |
| Tartrate of soda, - - - | 4.75 |
| A principle, eminently carbonized, probably altered cruor, - - | 31.40 |
| | 100.00 |

The analysis therefore shows the absence of fatty matter which is found in encephaloid productions, and exhibits a large proportion of all the materials of the blood.

*Structure.*—Much doubt still exists with regard to the nature of this product, and even whether it be a tissue. Laennec believed it to be an accidental tissue. If we examine melanosis in masses, no tissue-like appearance can be seen. Breschet could find no vessel, no nerve, no fibre, in these masses; he could not therefore agree with Laennec, Meckel, and others, in regarding it as a particular species of cancer, though he would probably admit that a cancerous mass might be infiltrated with melanotic fluid. I cannot subscribe to this opinion, though it be true that the black matter has been found in tissues disorganized to the last degree—though it has been observed in carcinoma of the eye. In all these cases there is, I suppose, only an accidental deposit of black matter in an accidental product. If this matter constituted one of the characters of cancer, we should regard as cancerous all parts where it is found deposited. Now what analogy is there between cancer and those black spots which we see on the peritoneum, the pleura, and the pericardium? Some persons regard it as a disease of the cellular tissue: no doubt melanotic matter is very commonly deposited in it. Many persons are of opinion that the black principle is an aberration of the pigment destined by nature to be deposited elsewhere, as the rete mucosum, the choroid, the hair; it is said that persons with light hair, and elderly persons whose hair no longer obtains the same quantity of colouring matter as it did in youth, as well as light gray or white horses, are most commonly the subjects of the disease; at the same time it must be admitted that it is by no means a universal rule. Beheir has seen melanotic matter in a nævus tumour; in this case tumours existed over the greater part of the surface, and the interior of the body, and the inclination on his mind was, that they were originally extravasations of blood, as in purpura; that they afterwards underwent particular changes; they were redder as they were more recent. Biett mentions another case in which an irritated nævus became the seat of similar deposition, which afterwards occurred in different parts of the body. I believe that this matter is the colouring and fibrinous portions of the blood in a particular state of alteration. Breschet pointed out its analogy with the choroid matter, that of the uvea, the placenta of some carnivora, the rete mucosum of the negro. He and Noack believe that the disease is a consequence of the accumulation in the blood of that carbon which was destined to colour certain tissues and organs. Assuming this as the most probable explanation of the formation of these tumours, it may be further assumed, that melanosis in masses is produced by the effusion of blood into the cellular tissues. Melanosis may affect most of our tissues and organs, the skin, the cellular system, the vessels, the lymphatic glands; Lobstein thought that the nerves were more surrounded than penetrated by it. Among the parenchymatous tissues, Fawdington, Halliday, Cullen and Carswell, have seen it affect the heart: the lungs, the liver, the pancreas, and the ovaries, are the most frequently affected. I am not aware of any case in which it has been deposited in the synovial membranes or articular cartilages. Halliday and Fawdington saw it affect many bones, Lobstein and Lauth have also seen examples: in a dead body dissected in this school last year it was found to affect many bones. The observations of Breshet and Cruveilhier, confirmed by those of Louth, seem to prove that this black matter had been found in blood vessels which had not been broken down. Treviranus, in some experiments on frogs, in which he interrupted the circulation in various ways, saw black, star-like points formed on the surface of many organs. Examined with a lens, these spots were found similar to the choroid pigment. In fact, it is most probable that melanosis is formed by the blood which has undergone a certain change; that the blood is even changed in the vessels themselves; that it is deposited in the tissue of organs by a kind of secretion analogous to that of the choroid and the black matter in the skin of the negro; from whence I conclude that it is not a sui generic pathological production, but a simple black deposit or colouring, sometimes of a healthy, sometimes of a diseased tissue, and sometimes of a tissue accidentally developed, such as a cancerous mass, and, therefore, that Laennec was in error in regarding it as a particular tissue.

*Symptoms.*—It seems questionable whether melanosis determines any special symptoms. Laennec believed it capable of gradually diminishing the vital powers, by altering the nutrition in parts of the body where it is deposited. Noack believes that in infiltrated melanosis the symptoms should be referred rather to the disease which has excited the infiltration. Still there are cases in which melanosis is the cause of the phenomena observed when the functions of organs are constrained, either by compression, as in the lung or the brain, or by acting as foreign bodies.

*Diagnosis.*—In the diagnosis two circumstances only may assist us where the disease is internal—black avacuations, which at last are indecisive, and the existence of melanotic spots, in, or immediately under the skin. When subcutaneous, if sufficiently superficial to show their colour, the diagnosis cannot be difficult.

*Prognosis.*—As to the prognosis, what I have said of the symptoms must at once lead to the conclusion that the disease is comparatively harmless, unless it interfere with the functions of organs, and then the gravity must depend upon the importance of the organ. If this interference does occur, the existence of melanosis is often not revealed until after death. Of course the danger is greater as the disposition to produce the disease is more decided, and subcutaneous tumours are usually conclusive evidence of the disposition.

*Causes.*—As to causes we can say little; reasoning from analogy, I believe that similar causes to those which produce purpura—causes capable of producing a relaxation of the solids and of thinning the fluids, may facilitate the production of this disease—and as to hereditary transmission, if proved in horses, it has never been made out in man.

*Treatment.*—In the treatment of this disease we have no fixed rules. Noack says that bleeding in summer has diminished the sizes of these tumours, and arrested their development. When few in number extirpation has succeeded. Damoiseau cured a case in this way; but unfortunately extirpation frequently does not prevent relapse, either at the same or another point. Gasparin says he has succeeded in preventing relapse by using sulphurous fumigation after extirpation. But the effects of these means must be further tested before they can be ranked as remedial agents.

### WOUNDS.

The violent action of all bodies harder than the tissues of our organs may overcome the resistance of those tissues, and produce a breach of surface or solution of continuity, or wound; for these are synonymous terms. If the solution of continuity affects the bony tissues it is termed a fracture.

Certain effects pretty uniformly accompany wounds, however they may be produced—pain, retraction of the edges, and hæmorrhage. The first varies with the quantity of sensibility with which the part is endowed. The second depends on the natural elasticity of the part—its extent being very variable; sometimes being scarcely sensible, at others, when the wound has implicated muscular fibres, being very great, especially when a long muscle is implicated, such as the sartorius, which may contract a third of its length. In fact, the longer the fibres the greater the retraction, the more numerous the fibres the more powerful the retraction. The third depends upon the section or wound of blood vessels; and as all organs admit a certain number of vessels into their texture, a wound can hardly happen without the shedding of blood.

*Union by "first intention."*—Left to itself, nature soon sets in motion the action necessary to repair the injury: that action is inflammation. The pain proceeding from the injury is increased by contact of air, and the necessary action is developed. If the wound be small, and the lips not far removed from each other, the inflammation is inconsiderable, serous fluid is effused, it gradually acquires more consistency, assumes the character of coagulable lymph, forms a medium between the lips, becomes organised, and union is complete. This semi-fluid substance may very soon be distinguished upon the surface of a wound, so soon, in fact, as the surfaces are sufficiently inflamed to coagulate the serous fluid which is at first effused. Between serous surfaces it has been seen in four hours, after twenty-four hours it is white and areolar, after forty-eight hours it is pervaded by blood canals, and by the sixth or seventh day it is completely organised. If the parts have been exactly brought together this bond of union or cicatrix is linear, fibro-cellular; its colour after a time is whiter, and its power of resistance greater than that of the adjoining tissues. In this way a ruptured continuity is restored, vessels pass through the cicatrix, though now and then difficulty is experienced in demonstrating them. Hunter, Wolf, and others, have shown that the vessels in a cicatrix are not simple extensions from the divided surfaces, but newly formed vessels which inosculate with the old ones. Many cases might be referred to in proof that nervous reparation also happens. Some years ago a young woman had neuralgia near the point of the little finger; many means were tried and failed; at last her medical attendant made a circular incision down to the bone; the neuralgia was dissipated, but by the end of two months it began again to be felt, and in six months it had acquired nearly its former intensity. Union so acquired was called by Hunter "union by first intention." To afford the greatest probability of such union it is necessary, or at least advisable, that both surfaces should be living. I say advisable, because the chances of success are greater; I believe it is unquestionable that parts entirely separated from the body will occasionally unite, but then they must be small parts, such as fingers. Again, a wound should not have been very long exposed, otherwise suppurative action may be established, and all chance of immediate union is dissipated.

Baronio completely detached from each side of the loins of a sheep a flap three inches by two; he substituted one for the other, and at the end of eleven days found both united; another time he allowed eighteen minutes, and another an hour to elapse, before they were applied but the results were the same. Wiseman made similar experiments with similar results. Hunter transplanted the testicles of a cock, as well as the spur and teeth of other animals, and they became equally adherent. For many years such things were not believed, but the examples are now too numerous and too well authenticated to admit of doubt: observations of ends of fingers separated and completely united have been recorded by Heister, Flurant, Piedagnel, Sommé, Busley, Balfour, Wigord, Houston, Bonn, and others; in one case the part has been detached an hour and a half. Examples of a similar kind implicating the nose are detailed by Garengeot, Blegny, Fiorarente, Molinelli, Leyseri, Loubet, Percy, Carlizzy. Portions of the ear by Laurent, Magnan. Burdach speaks of a case in which Lenhossek saw the ungual phalanx unite, Schopper saw two phalanges, Braun an entire finger, and adds that Marley and Lario had seen similar cases.

The precept which arises out of these cases is, that when the end of a finger, a nose, or an ear, is completely detached, if it be not much contused, if not more than three or four hours have elapsed since the accident, union should be attempted. In many cases it may be expected to fail, in a very few it may succeed. You must also bear in mind, that up to the present time these results have occurred only where very small portions of the body have been detached.

It is better also that there should be a similarity between the surfaces; but this is not indispensable: for instance, in excising a tumour, we remove a certain quantity of tissues, and similar parts do not come in contact, yet they heal by "first intention." The lips of the wound should not be severely contused, or suppurative action will most likely set in. Foreign bodies, such as a splinter, a ligature, a piece of linen, or any similar substance, will prevent immediate union, by setting up suppuration. A coagulum of blood, if thin, interposed between the lips of a wound, does not seem to constitute an obstacle to immediate union; Hunter, indeed, thought that "being endowed with life," it did not irritate; that the colouring and serous portions being removed, the coagulable lymph remained, was organized, and constituted the cicatrix," but in many cases it is hurtful. The general health and age of the patient, the period of the year, and a separation of the lips of the wound, may have material influence in producing or opposing union by first intention. With respect to temperature, Larrey, when in Egypt, was much struck with the rapidity with which wounds healed. Guyon and Breschet have been engaged at the Hotel Dieu in making experiments on this subject, which strengthen this belief, and it is, I think, certain that wounds heal more rapidly under warm water dressings than under ordinary treatment.

*Union by granulations.*—Want of proper contact, loss of substance, disorganizing contusion, and the presence of a foreign substance, may prevent union by first intention, may excite suppurative action. In this case, when the hæmorrhage is suspended for a certain time, as before, a serous exhalation continues, inflammation becomes more intense, too intense for serous exhalation; it is therefore suspended; the surface becomes comparatively dry, the lips are tumid, dry, and painful; by the third or fourth day, varying however with the vascularity of the tissue, the surface again becomes moist; a reddish fluid is effused, and a concrete or coagulated fluid soon covers it; small red elevations (granulations) become apparent, and upon their surface a creamy fluid-pus may be observed. As soon as this fluid is formed the inflammatory action subsides, the tumefaction of the edges is much lessened. Whether immediately before or immediately after (for it is a debated position) the secretion of pus, those granulations are covered by a membrane which affords their delicate tissue a certain protection against external violence; this tissue was called by Delpech the pyogenic tissue or membrane, and the term is now generally used. The granulations increase in number and in consistency; they, or the membrane which covers them, are endowed with considerable retractile powers, by which the surface of a wound is much lessened. These bodies may become too luxuriant, may sprout above the surrounding surface, but unless they rise very high, a pellicle spreads over and represses them. This covering is at first very thin and red, but gradually becomes very resistant, and losing its vascularity, becomes white. The duration of this process is of course very variable.

*Symptoms.*—These processes cannot take place in the economy without developing certain general or constitutional symptoms; they are less decided, as might be expected, in wounds which heal by first intention than in those which suppurate. In each case they must necessarily vary with the extent of the wound, and the excitability of the patient. When the wound is disposed to heal rapidly, or is not large, the general excitement is very inconsiderable; but if the local disturbance be great, there is restlessness, heat of skin, and quick pulse. When adhesion commences these symptoms rapidly subside: in all this, however, there must be great variety. If suppurative action set in, the general symptoms are better marked—this action is not developed without some febrile disturbance, which is declared on the second or third day; rigors are first observed; the skin is hot and clammy, the pulse quick, but not hard; the tongue is whitish. In ordinary cases this feverish action subsides after two or three days, the tongue cleans, thirst is abated, the appetite returns, and the disease is only local. If the patient be irritable and the wound large, the symptoms may be more serious, the feverish excitement may be more severe; there may be delirium, convulsions, spasms; these may be so great as to destroy life before suppurative action is established. If these first dangers be passed, all is not passed. If the wound be large, jagged, irregular, implicating important parts, phlebitis or other serious mischief may happen. The irritation of the wound may be so great, or the suppuration so abundant, that the system may give way under it.

*Forms of Wounds.*—A wound may be produced by many different agents; and these agents give peculiar characters to it. The agent may be a puncturing, incising, contusing, or lancinating instrument; it may be caused by projectiles from fire arms, by rabid or venomous animals, or it may be complicated by the insertion of peccant matter in the wound.

*Punctured Wounds.*—In a punctured wound only a very small extent of parts is divided, and those only corresponding to the point of the instrument; the rest are pushed aside, distended. Uusually the disturbance is not great, and the wound is soon cicatrized. But it is not always so; I have known an acupuncture-needle introduced into the muscles of the calf of the leg, followed by violent inflammation and abcess. It is rare, however, that a puncture is made under such advantageous circumstances as that in acupuncturation; the blow is often sudden and violent, the instrument is not always sharp-pointed. The orifice of such a wound is usually narrowest, narrower indeed than the instruments by which it is inflicted. You must not always expect that the orifice will have the form of the instruments which have made it: this is an important circumstance for you to bear in mind in giving evidence. If, in a dead body, a puncturing instrument, with cutting edges, be made to penetrate perpendicularly to the surface, the teguments being equally tense on all sides, the wound will fairly represent the form of the instrument; but if the same instrument penetrate obliquely, or if the teguments be not equally tense on all sides, the form of the wound will no longer represent that of the instrument. If the instrument be merely pointed, and not cutting, it is usually impossible from the form of the wound to judge of the shape of the instrument: even though the instrument be exactly rounded, and be plunged perpendicularly to the surface of the integuments, the wound may be oval or angular. Indeed, the same instrument plunged into the body in half a dozen different places, may produce wounds of as many different forms. If the punctured wound be large, it has this peculiarity; the symptoms are sometimes very serious, and apparently out of proportion with the extent of the wound. This is probably because the nervous filaments, are not fairly cut through, but torn apart. What gives colour to this belief is, that the painful effects of such injuries are often suddenly lessened by making the puncture an incision. Supposing this to be a fact, it is only to be explained in one of four ways; either the incision has laid open a purulent collection, or it causes the evacuation of extravasated matter, or completes the section of partially divided nervous filaments, or relieves strangulation, by cutting through fascia or aponeurosis. We know that the symptoms are similar to those resulting from puncture of nervous cords, and we know further that the complete section of the injured nerve often relieves the suffering. In this kind of wound the hæmorrhage is usually not great, except in a few cases where a large vessel is implicated. Almost always the tumefaction is greater than that which occurs in incised wounds. One important peculiarity belongs to them, it is the rapid diminution of the size of the wound; therefore it is unsafe to judge of the depth to which the instrument has penetrated by comparing it with the extent of the wound. For instance, if at half an inch from its point, the instrument is five lines wide, the wound measuring only three, you are not therefore to assume that the instrument has not penetrated to the extent of an inch or more. The consequences of a punctured wound may be very serious: it may penetrate into a cavity, it may wound an artery. But punctured wounds are most grave when a tissue is much intersected, like the palm of the hand and sole of the foot; inflammatory action is then often intense; they may also excite tetanus. I recollect a case of tetanus treated by M. Gaultier de Claubry, succeeding to a sting of a wasp in the sole of the foot, and another is mentioned where the patient trod on a needle. Sometimes the inflammation seems to be excited by strangulation, which may even proceed to gangrene.

*Prognosis.*—The prognosis of punctured wounds is generally more serious than of incised wounds. In incised wounds we less frequently see those accidents to which we have just referred. In cases of punctured wound of the scalp, erysipelas not unfrequently follows. When we know the accidents which may follow these wounds, we may anticipate them. If the wound be severe, and bleeds but little, we should endeavour to facilitate further bleeding: for instance, if the finger be punctured, by pressure and immersion in warm water we may often unload the vessels, and get rid of irritating substances.

*Treatment.*—The treatment to be employed in punctured wounds does not differ very much from that of incised wounds—cold local applications, careful dieting, and saline aperients. If the wound be a sword or other similar puncture, affecting the chest, although we believe it has not penetrated, it is necessary to bleed; if a similar wound penetrate a limb, again it is necessary to bleed; the quantity of blood to be regulated by the severity of the injury and the strength of the patient. If it be probable that a splanchnic cavity is penetrated, the bleeding must be large, and repeated upon the least appearance of re-action. As much as possible the patient should be placed in an inclined position, to admit of the drawing off of any fluid which the wound may contain. In these cases inflammation is sometimes developed in the wound, sometimes in the course of the lymphatics: these should be boldly treated by leeches; and it is a matter of great importance to place the part in such a position that the return of venous blood may be facilitated. In the limbs the part should be placed on a plane higher than the trunk. In cases where we cannot give the part a position which will facilitate the escape of pus, counter-openings are sometimes necessary. I must here urge you to be very careful in making counter-openings; though, apparently, a simple operation, it requires an accurate knowledge of anatomy. In such a case, so able an anatomist as Boyer once opened the profunda. Be very cautious, therefore, when it is necessary to make the openiug near an artery. Sometimes it will happen that though fluctuation was very evident, no pus follows the opening; you must then introduce a tent, place warm water dressing over it, and very likely in a day or two the pus will flow through the opening. If a foreign substance be contained in the wound, it should be extracted, if superficial or easily removed; if not, if the foreign body be not likely to excite irritation, it may be left until suppuration has loosened it. In some cases, suction is useful in withdrawing blood or other substance; but, instead of suction, with all its mummeries, we employ, in the present day, cupping glasses. If used, it must be done prudently, lest by removing clots they occasion hæmorrhage. La Motte and John Bell were strong advocates for their employment.

*Incised Wounds.*—A fair incised wound is made by an instrument capable of cutting through our tissues without contusing them. Such a wound must be very variable; it may be a simple incision without loss of substance, or the instrument may cut out a portion of our tissues, and of such an extent as to prevent the sides from being brought together; this happens sometimes when a flap is taken from the forehead to make a new nose. The part may not be completely cut away, but may be attached by a pedicle; that pedicle may be large enough to maintain vitality in the flap, or it may not; in the latter case, it may be gangrened. The wound may implicate the skin, cellular tissue, and muscular fascia—it may extend to the muscles. It may be parallel to the muscular fibres; then there is little gaping of the wound; it may be oblique or perpendicular to the course of the fibres, and the sides of the wound may be then much retracted. It may implicate important blood vessels, and there may be considerable hæmorrhage. Sometimes considerable vessels are cut through without hæmorrhage—in certain amputations, in extirpation of the breast, there is occasionally spontaneous cessation of hæmorrhage. This circumstance is owing, I apprehend, to a moral impression, to pain, to a general spasmodic contraction; perhaps, to some extent, to the contraction of the newly incised tissues upon the vessels. In a few hours this state is dissipated, and hæmorrhage will occur; therefore, in such cases, be upon your guard. The instrument may cut through nervous cords, and the parts to which these nerves proceed may loose sensation and motion. The nerve may be incompletely divided, sensation may remain intact, and motion is not destroyed, but acute pains are felt. It may penetrate to the periosteum, may wound it: sometimes this is not a serious complication, but caries or necrosis may be a consequence. It may implicate the bony tissues, cutting more or less completely through the bone. These wounds are, therefore, in many cases, complex; varying infinitely in their character and their gravity. Their gravity may be modified by age; they are dangerous generally at the two extremes of life; in children the hæmorrhage is often great; in old age adhesion is with difficulty set up. Incised wounds, whether simple or complicated, occasion certain pretty constant symptoms—*Pain* of variable severity, increased by exposure, lessened when the sides of the wound are brought together. *Hæmorrhage*, which is almost a necessary consequence of all wounds: this may be trifling or it may be considerable; if a tolerably large vessel be not divided it often ceases of itself, becoming more and more serous, until the orifices are blocked up by clots. *Separation of the edges of the wound.* This may depend upon the ordinary elasticity and contractility of tissues, but it may be owing to peculiar situation—supposing a wound of the front of the thigh, cutting through the extensor muscles, it is evident that the position which you give the limb will increase or diminish the separation; if you flex the thigh upon the pelvis, you will lessen it; if you extend the thigh, you will increase it.

Almost always, soon after the action of the cutting instruments, the lips of the wound become slightly tumefied. This is a consequence of pain, and without it union would not take place—it is therefore salutary; but it may become so violent as to prevent immediate union, and may determine suppuration. Exempt from complication and left to itself, I have already described the course which nature takes in small wounds; but when larger the separation increases, and suppuration is almost inevitable, unless art interfere. Even when the most careful treatment is employed this may happen. However extensive the incision, and however nearly a part may be detached from the body, we must not despair of seeing it united; the point of attachment may be insufficient to support life in the part, but of this you can never be certain, because a part may be completely detached, and yet may unite. We have known a finger hanging by a single tendon, put in contact and united; the nose has adhered only by an extremely fine point, brought into apposition with the part from which it has been cut away, and united. An ear, a finger, a nose, has been completely detached, and afterwards united. These examples, it is true, refer principally to the fingers and toes. Can we, from this evidence, infer that a part cut from the back may afterwards unite? It is possible, but I know no direct evidence in support of it, except that of Baronio, in sheep.—*Lond. Med. Gaz.*

---

*Case in which Tracheotomy was performed for the removal of a Foreign Body in the Air Passages. By* Benjamin Travers, *Esq., Jun. Communicated by* Benjamin Travers, *Esq., F. R. S., &c. &c.*—A child, about six years of age, while seated on the ground, eating cherries, was suddenly thrown backwards, and immediately seized with a violent fit of choking, and every symptom of impending suffocation, a condition which is said to have lasted a full hour. This accident was followed by spasmodic pain in the chest, dyspnœa, and other symptoms of acute inflammation; but the cough ceased so entirely for a considerable time, that the surgeon in attendance concluded that the offending body had passed down the œsophagus, and not into the air-tube. The symptoms, however, recurring with great violence, Mr. Travers was called to see the patient, and at his first visit, two or three weeks after the accident, found the breathing stridulous and laboured, the pulse small and hurried, the countenance suffused and anxious; there were frequent paroxysms of croupy cough, with much consequent exhaustion. The author opened the tracheal tube, between the isthmus of the thyroid gland and the top of the sternum, to the immediate relief of the patient's breathing; and the cough did not return again for some days. Before leaving the patient the author passed a silver catheter upwards through the larynx, with a view of ascertaining that the tube was not obstructed in that direction. Six or seven weeks after the operation the wound was allowed to heal, when the cough shortly re-appeared, with night-sweats and loss of appetite. About a month after the closure of the wound the cherry-stone, which had passed into the tube, was ejected during a violent fit of coughing, accompanied by a small quantity of pus.—*Med. and Chirurg. Soc.*

*London Lancet.*

